# Repeated Access to Patient Portal While Awaiting Test Results and Patient-Initiated Messaging

**DOI:** 10.1001/jamanetworkopen.2025.4019

**Published:** 2025-04-08

**Authors:** Bryan D. Steitz, Robert W. Turer, Liz Salmi, Uday Suresh, Scott MacDonald, Catherine M. DesRoches, Adam Wright, Jeremy Louissaint, S. Trent Rosenbloom

**Affiliations:** 1 Department of Biomedical Informatics, Vanderbilt University Medical Center, Nashville, Tennessee; 2 Department of Emergency Medicine, UT Southwestern Medical Center, Dallas, Texas; 3 Clinical Informatics Center, UT Southwestern Medical Center, Dallas, Texas; 4 Department of Medicine, Beth Israel Deaconess Medical Center, Boston, Massachusetts; 5 Department of Clinical Informatics, University of California, Davis Health, Sacramento; 6 Department of Medicine, Harvard Medical School, Boston, Massachusetts; 7 Division of Digestive and Liver Diseases, UT Southwestern Medical Center, Dallas, Texas; 8 Department of Medicine, Vanderbilt University Medical Center, Nashville, Tennessee; 9 Department of Pediatrics, Vanderbilt University Medical Center, Nashville, Tennessee

## Abstract

**Importance:**

Patients have immediate access to test results in the patient portal, which can cause worry. Identifying behaviors associated with worry while awaiting results may allow health systems to support patients and reduce message volumes.

**Objective:**

To describe characteristics of patients who refresh their portal while awaiting test results and measure the association between refresh behavior and patient-initiated messaging.

**Design, Setting, and Participants:**

This retrospective cross-sectional study was performed at Vanderbilt University Medical Center, which uses a MyChart-based patient portal. All adult patients (aged ≥18 years) who received outpatient test results between January 1, 2022, and December 31, 2023, were included.

**Exposures:**

Use of the patient portal to review test results.

**Main Outcomes and Measures:**

The primary outcome was the proportion of patients who refreshed the portal while awaiting test results. Tests were stratified into low-sensitivity (eg, basic metabolic panel) and high-sensitivity (eg, tissue biopsy) groups using validated categories based on each test’s likelihood of being misinterpreted or causing distress. Portal access logs were used to identify refresh behaviors, in which patients accessed the portal seeking new results. Secondary outcomes included the association between patient characteristics and refresh behavior and between refresh behavior and messaging as measured using multivariable logistic regression.

**Results:**

A total of 968 774 results were reviewed by 290 349 patients (mean [SD] age, 47.8 [18.0] years; 66.3% female). Patients refreshed their portal for 25.9% of results. Patients more commonly refreshed for high-sensitivity results (25 280 of 64 356 [39.3%]) compared with low sensitivity results (225 516 of 904 418 [24.9%]) (*P* < .001). Patients who enabled notifications had higher odds of refreshing for high-sensitivity results (odds ratio, 1.11; 95% CI, 1.06-1.17) and low-sensitivity results (odds ratio, 1.54; 95% CI, 1.49-1.59). Each refresh for high-sensitivity results was associated with a lower increase in the probability of messaging within 24 hours (mean marginal effect, 0.41; 95% CI, 0.34-0.49) compared with refreshing for low-sensitivity results (mean marginal effect, 1.03; 95% CI, 0.96-1.11).

**Conclusion and Relevance:**

This cross-sectional study found an association of patients’ refresh activity for low-sensitivity results with patient-initiated messaging after review. These findings suggest that refresh behavior, a potential measure of worry, may be a characteristic of patient attitudes and preferences rather than the test.

## Key Points

**Question:**

Among adult patients who receive outpatient tests, what proportion of patients refresh their portal in anticipation of results?

**Findings:**

In this cross-sectional study of 290 349 patients who reviewed 968 774 results between 2022 and 2023, patients refreshed their portal for 25.9% of results. Patients more commonly refreshed for highly sensitive results, but refresh behavior while awaiting nonsensitive results was associated with a higher probability of patient-initiated messaging within 24 hours.

**Meaning:**

These findings suggest that refresh behavior, a possible measure of worry, may be a characteristic of patient attitudes and preferences, rather than results sensitivity, that may contribute most substantially to message-related clinical workload.

## Teaser

This cross-sectional study examines the characteristics of outpatients who refresh their portal while awaiting test results and the association between refresh behavior and patient-initiated messaging.

## Introduction

The 21st Century Cures Act mandates that patients have immediate electronic access to their test results upon request as soon as they become available in the electronic health record. Many health systems comply by making results available in a patient portal.^1^ Before the Cures Act, health systems typically released a subset of results through the portal.^2^ Results considered potentially emotionally sensitive or difficult to interpret were commonly withheld or released after a delay. These delays were intended to give clinicians time to review results, formulate a management plan, and follow up with patients about findings before patients saw the results. Now that results are immediately available, more than 40% of results classified as sensitive are reviewed by patients before their clinicians, which has been associated with a doubling in patient-initiated messages on the portal.^3,4,5^ Most portal users are satisfied with receiving test results online^6^ and prefer to receive results as soon as they become available, but some patients have reported increased worry.^7,8^

Patients often experience worry or anxiety while awaiting and reviewing results from clinical testing, especially when findings may indicate a potentially life-changing diagnosis.^6,9,10,11^ Heightened worry is prevalent when patients review results that are difficult to interpret or contextualize.^6,9^ Resources have been developed to help patients review results, including test-specific informational content and education modules.^12,13^ Many patients have reported that heightened worry while awaiting results often exceeds the stress associated with receiving the result, even when findings indicate bad news.^14,15,16,17^ Some organizations have prioritized precounseling when tests are ordered to prepare patients about possible findings and expectations for follow-up,^18^ but it is unclear whether these initiatives have been successful in minimizing worry.^7^ Now that patients have immediate access to results, there is concern that the ability to repeatedly check for anticipated results may exacerbate worry.

Identifying patients who exhibit behaviors associated with worry while awaiting results may allow health systems to proactively support patients, improve the patient experience, and reduce message volumes. Data from portal access logs may provide granular insight into behaviors in anticipation of new results. We used portal access logs to identify refresh behaviors in which patients repeatedly access the portal while seeking new results. We quantified the prevalence of refresh behavior, investigated characteristics of patients who refresh for results, and measured the association between refresh activity and messaging.

## Methods

### Study Design and Setting

This cross-sectional study was conducted at Vanderbilt University Medical Center (VUMC), a large academic medical center in Nashville, Tennessee, that delivers primary and tertiary care to patients across the southeastern US. Patients at VUMC can review test results and message their clinicians through My Health at Vanderbilt (MHAV), VUMC’s MyChart-based patient portal (Epic Systems Corp).^2^ The Vanderbilt University Institutional Review Board approved the study procedures and granted a waiver of informed consent because the research was deemed less than minimal risk and was conducted on data previously collected during routine patient care. We followed the Reporting of Studies Conducted Using Observational Routinely Collected Health Data (RECORD) reporting guideline.^19^

We studied test results ordered during ambulatory encounters and released to adult patients (aged ≥18 years) through MHAV between January 1, 2022, and December 31, 2023. During the study, all results were immediately released to the portal as soon as they became available in the electronic health record. We defined test sensitivity using locally curated and validated categories that guided delayed result release timing prior to the Cures Act.^2^ These historical delays were based on each test’s likelihood of being misinterpreted or causing emotional distress. Historical sensitivity categories included tests that were released immediately (eg, basic metabolic panel) or had a delayed release after 1 day (eg, thyroid function tests), 3 days (eg, radiology reports), 7 days (eg, testing for sexually transmitted infections), or 14 days (eg, anatomic pathology or cytology reports). To avoid overcounting refresh behaviors for results released in proximity, we collapsed results released within a 6-hour window into a single result group and retained the latest release time and all historical release classifications. To avoid misclassifying refresh behavior in instances for which results from multiple historical release categories were released during the same time window, we only analyzed result groups in which all tests were from the immediate release category (low sensitivity) or the 14-day release category (high sensitivity). This strategy excluded 6617 result groups but allowed us to focus our comparison on highly sensitive vs nonsensitive results. Example tests within each category are available in eTable 1 in Supplement 1. We extracted study data, including patient sociodemographic characteristics, test result details, and portal access logs from Epic’s Clarity reporting database.

### Measures

For each high-sensitivity and low-sensitivity result group, we collected patient sociodemographic variables, result details, a count of messages sent following result review, and portal access logs corresponding to any accessed feature in the time between specimen collection and result release. Sociodemographic variables included age at first-ordered test, legal sex, race, ethnicity, preferred language, and insurance. Given the growing reliance on portals to deliver health information, it is crucial to understand whether disparities exist in the ways that patients interact with their test results. We grouped age into the following categories: 18 to 34 years, 35 to 49 years, 50 to 64 years, 65 to 84 years, and 85 years or older. We included race and ethnicity variables as documented in the electronic health record. Race categories included American Indian or Alaska Native, Asian, Black or African American (hereafter, Black), Middle Eastern or North African, Native Hawaiian or Pacific Islander, White, and other (including none of these, other, prefer not to answer, and unable to provide). Ethnicity variables included Hispanic or Latino (hereafter, Hispanic), non-Hispanic or Latino (hereafter, non-Hispanic), and other (including unknown, prefer not to answer, or unable to provide). During the study, MHAV was available in English and Spanish, so we reported preferred language as English, Spanish, or other (eg, Arabic, Japanese, Somali, Vietnamese). We classified insurance as commercial (including commercial, agency, exchange, and managed care), Medicare, Medicaid, uninsured, and other (including workers’ compensation or other nonclassified payers).

Details of results released within each group included the time of specimen collection, time of result release, whether patients enrolled in notifications, and whether the result was classified as low sensitivity or high sensitivity. During the study, text messages, email, and MyChart mobile application–based notifications were turned off by default for new results,^3^ but patients could manually configure notifications at their discretion. For each result group, we counted the number of tests ordered within the same day. We also evaluated whether a patient initiated a portal message within 24 hours after review of the last result per group as a dichotomous variable.

We used portal access logs to identify refresh behavior in which a patient or designated proxy accessed the portal to check for new results. Portal access logs contained a time-stamped sequence of actions performed in MHAV. We grouped portal activity into sessions, defined as a sequence of contiguous activity performed by a user within 5 minutes of a prior action. Within each session, we identified refresh behavior as reviewing the list of available results without subsequently accessing result details. For each result group, we calculated the refresh count as the number of sessions containing at least 1 refresh behavior in the time between specimen collection and result release to the patient portal. This measure of refresh behavior enabled us to identify the number of times a user returned to the portal to check for new results.

### Statistical Analysis

We calculated descriptive statistics stratified by whether patients refreshed for results. We compared sociodemographic characteristics by result review and refresh activity using a χ^2^ test for categorical variables and a 1-way analysis of variance for continuous variables. We tested for statistically significant differences in refresh behavior and messaging between the high- and low-sensitivity result groups using 2-tailed *t* tests with unequal variances.

We fit 2 exploratory multivariable logistic regression models to evaluate the association between patient and result characteristics and frequent refreshing. We defined frequent refreshing as a binary variable indicating that patients refreshed more than the population mean number of refreshes. We also fit multivariable logistic regression models to characterize adjusted differences in patient-initiated messaging as a function of refresh counts. Odds ratios (ORs) from the model evaluating messaging were converted to mean marginal effects. All models controlled for patient characteristics, including age, sex, ethnicity, race, language, insurance, years enrolled in MHAV, duration between specimen collection and result release (result processing time), whether patients enabled notifications for new results, and the number of ordered tests per day. Results are reported at the granularity of a result group. For each model, we computed standard errors, clustered by patient, to account for patients with multiple result groups.

We set α to .05 for significance testing. All analyses were performed using R, version 4.3.3 (R Foundation).

## Results

During the 2-year study period, we observed 329 317 patients who received 1 208 205 results. Overall, 290 349 patients (88.2%; mean [SD] age at first-ordered test result, 47.8 [18.0] years; 66.3% female and 33.7% male; 0.4% reported as American Indian or Alaska Native, 1.9% as Asian, 12.5% as Black, 0.3% as Middle Eastern or North African, 0.1% Native Hawaiian or Pacific Islander, 77.9% White, and 6.9% other or unknown race; and 4.9% reported as Hispanic, 84.9% as non-Hispanic, and 10.1% as other or unknown ethnicity) reviewed 968 774 results (80.2%) (Table 1). Results were most reviewed White (79.2% compared with 2.0% Asian, 11.3% Black, and 6.7% other or unknown race), non-Hispanic (85.4% compared with 4.8% Hispanic and 9.8% other or unknown ethnicity), and English-speaking (98.5%) patients who were enrolled in the portal a mean (SD) of 2.4 (1.8) years before receiving their first test result (Table 2).

**Table 1.** Characteristics of Patients Who Received and Reviewed Test Results Via the Patient Portal

Patient characteristic	Test results, No. (%)^a^				*P* value
	Never viewed (n = 239 431)	Viewed without refresh (n = 717 978)	Viewed and refreshed (n = 250 796)	All (N = 1 208 205)	
Age group, y					
18-34	46 340 (19.4)	176 343 (24.6)	66 391 (26.5)	289 074 (23.9)	<.001
35-49	43 248 (18.1)	159 775 (22.3)	60 573 (24.2)	263 596 (21.8)	
50-64	64 039 (26.7)	188 225 (26.2)	66 855 (26.7)	319 119 (26.4)	
65-84	78 984 (33.0)	183 711 (25.6)	54 692 (21.8)	317 387 (26.3)	
≥85	6820 (2.8)	9924 (1.4)	2285 (0.9)	19 029 (1.6)	
Sex					
Female	143 355 (59.9)	471 884 (65.7)	174 171 (69.4)	789 410 (65.3)	<.001
Male	96 076 (40.1)	246 094 (34.3)	76 625 (30.6)	418 795 (34.7)	
Ethnicity					
Hispanic or Latino	12 656 (5.3)	35 204 (4.9)	11 570 (4.6)	59 430 (4.9)	<.001
Not Hispanic or Latino	198 851 (83.1)	609 765 (84.9)	217 534 (86.7)	1 026 150 (84.9)	
Other or unknown^b^	27 924 (11.7)	73 009 (10.2)	21 692 (8.6)	122 625 (10.1)	
Race					
American Indian or Alaska Native	841 (0.4)	2838 (0.4)	1051 (0.4)	4730 (0.4)	<.001
Asian	3335 (1.4)	14 411 (2.0)	5294 (2.1)	23 040 (1.9)	
Black or African American	41 127 (17.2)	84 736 (11.8)	25 022 (10.0)	150 885 (12.5)	
Middle Eastern or North African	789 (0.3)	2354 (0.3)	767 (0.3)	3910 (0.3)	
Native Hawaiian or Pacific Islander	206 (0.1)	623 (0.1)	196 (0.1)	1025 (0.1)	
White	174 282 (72.8)	563 743 (78.5)	203 215 (81.0)	941 240 (77.9)	
Other or unknown^c^	18 851 (7.9)	49 273 (6.9)	15 251 (6.1)	83 375 (6.9)	
Preferred language					
English	230 775 (96.4)	706 044 (98.3)	247 802 (98.8)	1 184 621 (98.0)	<.001
Spanish	4970 (2.1)	5929 (0.8)	1483 (0.6)	12 382 (1.0)	
Other^d^	3686 (1.5)	6005 (0.8)	1511 (0.6)	11 202 (0.9)	
Insurance					
Commercial	114 042 (47.6)	437 342 (60.9)	160 056 (63.8)	711 440 (58.9)	<.001
Medicaid	14 262 (6.0)	34 658 (4.8)	12 486 (5.0)	61 406 (5.1)	
Medicare	52 873 (22.1)	124 793 (17.4)	41 207 (16.4)	218 873 (18.1)	
Uninsured	6276 (2.6)	12 449 (1.7)	3350 (1.3)	22 075 (1.8)	
Other^e^	51 978 (21.7)	108 736 (15.1)	33 697 (13.4)	194 411 (16.1)	
Years enrolled in portal					
Mean (SD)	2.0 (1.8)	2.3 (1.8)	2.4 (1.8)	2.3 (1.8)	<.001
Median (IQR)	1.7 (0.0-4.2)	2.2 (0.4-4.2)	2.4 (0.5-4.2)	2.1 (0.3-4.2)	
Notified of result					
No	188 060 (78.5)	396 365 (55.2)	102 897 (41.0)	687 322 (56.9)	<.001
Yes	51 371 (21.5)	321 613 (44.8)	147 899 (59.0)	520 883 (43.1)	
Patient-initiated message within 24 h					
No	224 592 (93.8)	618 989 (86.2)	197 815 (78.9)	1 041 396 (86.2)	<.001
Yes	14 839 (6.2)	98 989 (13.8)	52 981 (21.1)	166 809 (13.8)	
No. of ordered results					
Mean (SD)	4.2 (3.9)	4.2 (3.6)	6.8 (5.1)	4.7 (4.1)	<.001
Median (IQR)	3.0 (1.0-6.0)	3.0 (1.0-6.0)	6.0 (3.0-10.0)	4.0 (2.0-7.0)	

^a^ Summary statistics are calculated per group of test results released within a 6-hour window.

^b^ Includes unknown, prefer not to answer, or unable to provide as indicated in the electronic health record.

^c^ Includes none of these, other, prefer not to answer, and unable to provide as indicated in the electronic health record.

^d^ Includes, for example, Arabic, Japanese, Somali, and Vietnamese.

^e^ Includes workers’ compensation and nonclassified payers.

**Table 2.** Characteristics of Patients Who Reviewed Test Results Via the Patient Portal, Stratified by Sensitivity Category

Patient characteristic	Test results, No. (%)^a^			*P *value
	Low sensitivity (n = 904 418)	High sensitivity (n = 64 356)	All (N = 968 774)	
Age group, y				
18-34	216 241 (23.9)	26 493 (41.2)	242 734 (25.1)	<.001
35-49	201 565 (22.3)	18 783 (29.2)	220 348 (22.7)	
50-64	241 841 (26.7)	13 239 (20.6)	255 080 (26.3)	
65-84	232 778 (25.7)	5625 (8.7)	238 403 (24.6)	
≥85	11 993 (1.3)	216 (0.3)	12 209 (1.3)	
Sex				
Female	587 397 (64.9)	58 658 (91.1)	646 055 (66.7)	<.001
Male	317 021 (35.1)	5698 (8.9)	322 719 (33.3)	
Ethnicity				
Hispanic or Latino	42 947 (4.7)	3827 (5.9)	46 774 (4.8)	<.001
Not Hispanic or Latino	772 177 (85.4)	55 122 (85.7)	827 299 (85.4)	
Other or unknown^b^	89 294 (9.9)	5407 (8.4)	94 701 (9.8)	
Race				
American Indian or Alaska Native	3657 (0.4)	232 (0.4)	3889 (0.4)	<.001
Asian	18 109 (2.0)	1596 (2.5)	19 705 (2.0)	
Black or African American	102 824 (11.4)	6934 (10.8)	109 758 (11.3)	
Middle Eastern or North African	2925 (0.3)	196 (0.3)	3121 (0.3)	
Native Hawaiian or other Pacific Islander	762 (0.1)	57 (0.1)	819 (0.1)	
White	717 052 (79.3)	49 906 (77.5)	766 958 (79.2)	
Other or unknown^c^	59 089 (6.5)	5435 (8.4)	64 524 (6.7)	
Preferred language				
English	890 738 (98.5)	63 108 (98.1)	953 846 (98.5)	<.001
Spanish	6726 (0.7)	686 (1.1)	7412 (0.8)	
Other language^d^	6954 (0.8)	562 (0.9)	7516 (0.8)	
Insurance				
Commercial	547 935 (60.6)	49 463 (76.9)	597 398 (61.7)	<.001
Medicaid	41 501 (4.6)	5643 (8.8)	47 144 (4.9)	
Medicare	161 971 (17.9)	4029 (6.3)	166 000 (17.1)	
Uninsured	15 061 (1.7)	738 (1.1)	15 799 (1.6)	
Other^e^	137 950 (15.3)	4483 (7.0)	142 433 (14.7)	
Years enrolled in portal				
Mean (SD)	2.4 (1.8)	2.1 (1.8)	2.4 (1.8)	<.001
Median (IQR)	2.3 (0.4-4.2)	1.9 (0.1-4.2)	2.3 (0.4-4.2)	
Refreshed for result				
No	678 902 (75.1)	39 076 (60.7)	717 978 (74.1)	<.001
Yes	225 516 (24.9)	25 280 (39.3)	250 796 (25.9)	
Notified of result				
No	473 981 (52.4)	25 281 (39.3)	499 262 (51.5)	<.001
Yes	430 437 (47.6)	39 075 (60.7)	469 512 (48.5)	
Patient-initiated message within 24 h				
No	759 165 (83.9)	57 639 (89.6)	816 804 (84.3)	<.001
Yes	145 253 (16.1)	6717 (10.4)	151 970 (15.7)	
No. of ordered results				
Mean (SD)	5.0 (4.2)	3.4 (3.5)	4.9 (4.2)	<.001
Median (IQR)	4.0 (2.0-7.0)	2.0 (1.0-4.0)	4.0 (2.0-7.0)	

^a^ Summary statistics are calculated per group of test results released within a 6-hour window.

^b^ Includes unknown, prefer not to answer, or unable to provide as indicated in the electronic health record.

^c^ Includes none of these, other, prefer not to answer, and unable to provide as indicated in the electronic health record.

^d^ Includes, for example, Arabic, Japanese, Somali, and Vietnamese.

^e^ Includes workers’ compensation and nonclassified payers.

Table 1 presents patient demographics stratified by result review and refresh activity. There were 272 103 patients (93.7%) who reviewed 904 418 results (93.4%) classified as low sensitivity and 53 234 patients (18.3%) who reviewed 64 356 results (6.6%) classified as high sensitivity. Demographic characteristics of patients who reviewed results stratified by sensitivity category are presented in Table 2. We observed refresh behavior among 108 056 patients (37.2%) for 250 796 results (25.9%), with a mean (SD) of 2.5 (3.5) refreshes per result group. Result groups in the fifth percentile of refresh behavior had 7 refreshes, and the first percentile had 16 refreshes. Patients more commonly refreshed for high-sensitivity results (39.3%) vs low-sensitivity results (24.9%) (*P* < .001). Details of result review and refresh patterns stratified by sensitivity are presented in eTable 2 in Supplement 1.

**Figure 1.**
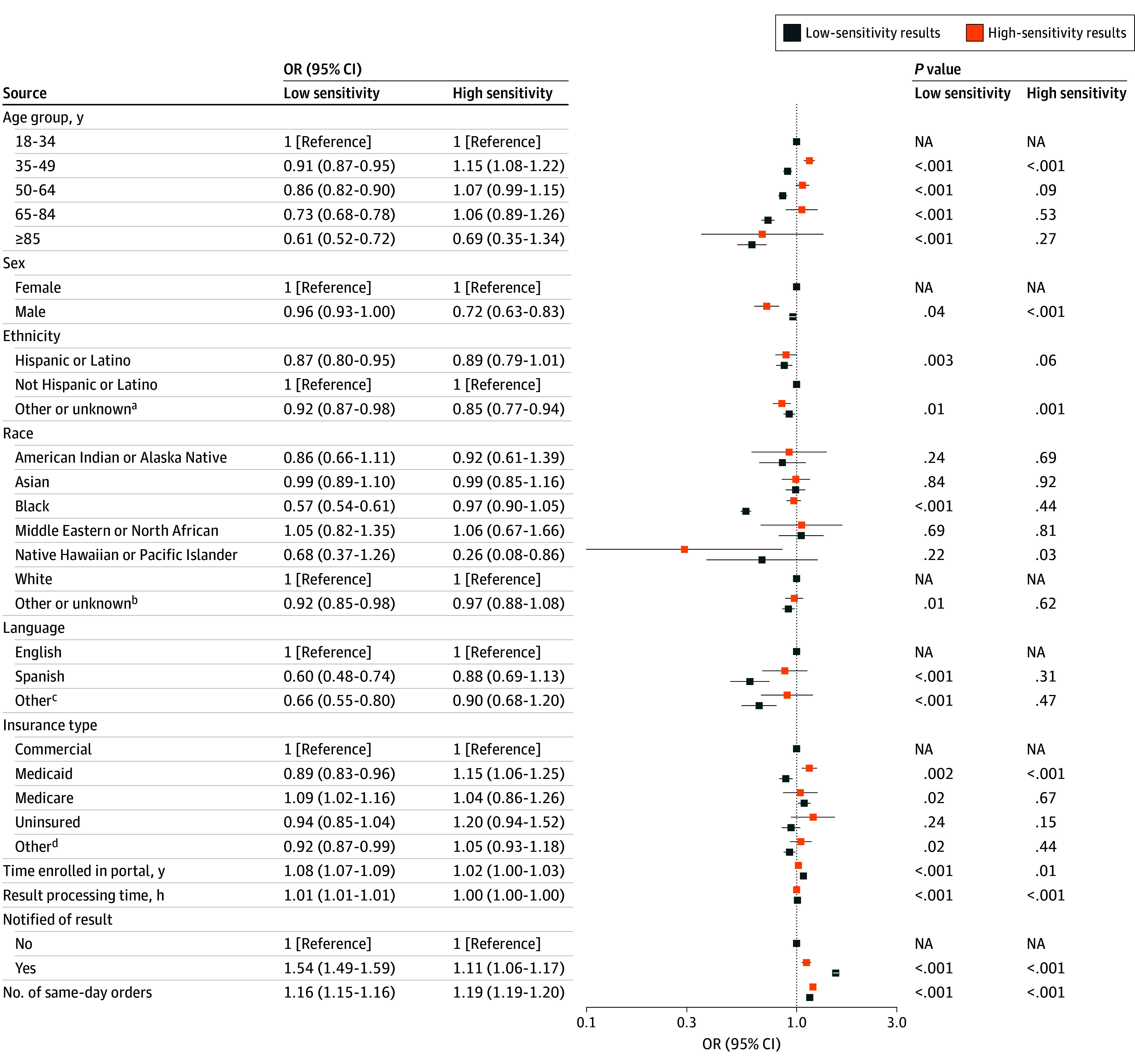
Odds of Frequent Refreshing for Reviewed Test Results, Stratified by Test Result Sensitivity OR indicates odds ratio. ^a^Includes unknown, prefer not to answer, or unable to provide as indicated in the electronic health record. ^b^Includes none of these, other, prefer not to answer, and unable to provide as indicated in the electronic health record. ^c^Includes, for example, Arabic, Japanese, Somali, and Vietnamese. ^d^Includes workers’ compensation and nonclassified payers.

Figure 1 depicts model findings describing the association between patient and test characteristics with frequent refreshing (≥3 refreshes). Patients who enabled notifications had higher odds of frequent refresh behavior for high-sensitivity results (OR, 1.11 [95% CI, 1.06-1.17]) and low-sensitivity results (OR, 1.54 [95% CI, 1.49-1.59]). Across both high-sensitivity and low-sensitivity results, female patients had higher odds of frequent refreshing compared with male patients (OR, 0.96 [95% CI, 0.93-1.00] and 0.72 [95% CI, 0.63-0.83], respectively). Younger, White, non-Hispanic, and English-speaking patients were more likely to exhibit frequent refreshing compared with patients who were older (aged 50-64 years: OR, 0.86 [95% CI, 0.82-0.90]; aged 65-84 years: OR, 0.73 [95% CI, 0.68-0.78]; aged ≥85 years: OR, 0.61 [95% CI, 0.52-0.72]), of Hispanic ethnicity (OR, 0.87 [95% CI, 0.80-0.95]), or non–English-speaking (Spanish: OR, 0.60 [95% CI, 0.48-0.74]; other language: OR, 0.66 [95% CI, 0.55-0.80]), but these associations were not significant among high-sensitivity results. Patients who had Medicaid insurance had lower odds of frequent refreshing for low-sensitivity tests (OR, 0.89 [95% CI, 0.83-0.96]) but higher odds of frequent refreshing for high-sensitivity tests (OR, 1.15 [95% CI, 1.06-1.25]) compared with those with commercial insurance. A longer time enrolled in the portal and more same-day orders were associated with higher odds of frequent refreshing for both high-sensitivity tests (OR, 1.02 [95% CI, 1.00-1.03] and 1.19 [95% CI, 1.19-1.20], respectively) and low-sensitivity tests (OR, 1.08 [95% CI, 1.07-1.09] and 1.16 [95% CI, 1.15-1.16], respectively).

**Figure 2.**
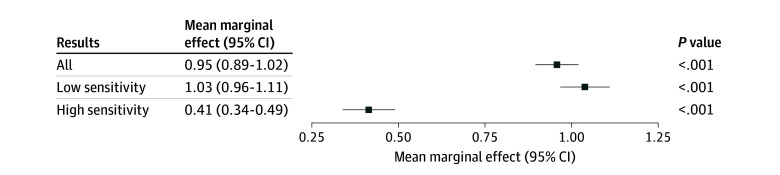
Marginal Effects of Refresh Activity on Patient-Initiated Messaging Within 24 Hours of Result Review Models adjust for age group, sex, ethnicity, race, insurance type, time enrolled in portal at result release, result processing time, whether patients enrolled in notifications for new results, and the number of tests ordered per day.

Patients who refreshed for results sent more messages within 24 hours of result review compared with patients who did not refresh (52 981 of 250 796 [21.1%] vs 98 989 of 717 978 [13.8%]; *P* < .001) (Table 1). Among patients who refreshed, patients more frequently messaged within 24 hours after reviewing low-sensitivity results (49 717 of 225 516 [22.0%]) than high-sensitivity results (3264 of 25 280 [12.9%]) (*P* < .001) (eTable 2 in Supplement 1). Multivariable models of patient-initiated messaging as a function of refresh activity found that each refresh was associated with an increase in the probability of messaging within 24 hours of review (mean marginal effect, 0.95; 95% CI, 0.89-1.02; *P* < .001) (Figure 2). Results from the unadjusted model are presented in eTable 3 in Supplement 1. Refreshes for low-sensitivity results had a larger change in the probability of messaging within 24 hours than for high-sensitivity results (mean marginal effect, 1.03 [95% CI, 0.96-1.11] vs 0.41 [95% CI, 0.34-0.49], respectively).

## Discussion

This cross-sectional study of more than 1 million test results found that patients commonly return to the portal multiple times (ie, refreshing) while waiting for results to become available. We observed refresh behavior among 37.2% of patients who reviewed results, including 39.3% of patients who reviewed high-sensitivity results. Our analysis of messaging trends found that frequent refreshing while awaiting results was associated with a higher probability of initiating a message after results review. Refresh activity for low-sensitivity results was more commonly associated with messaging. This finding suggests that frequent refreshing, a possible surrogate measure of patient worry about a result, may be a characteristic of patient attitudes and preferences rather than of the test result itself.

Awaiting uncertain news and reviewing results are well-documented causes of medical anxiety and worry.^9,14,15^ Several studies have investigated the association of result release with patient worry and psychological distress. Patients experienced heightened worry when results were abnormal,^6,7,20^ highly sensitive,^11,21^ or difficult to interpret.^22^ Fewer studies have investigated feelings of worry while awaiting results, despite research indicating that awaiting uncertain news often contributes to greater anxiety than receiving the news.^14,15^ We hypothesize that measuring refresh behavior while awaiting results may offer insights into potentially anxious behavior across a broad cohort. Our approach to measuring refresh activity was enabled by portal access logs, which may offer an opportunity to detect refresh behavior and allow for intervention in real time. Further research is necessary to quantify the association between worry and refresh activity.

Our multivariable models identified patient and test characteristics associated with frequent refreshing. We noted the greatest odds of frequent refreshing among younger, non-Hispanic, and English-speaking patients, mirroring the largest group of portal users.^23,24,25^ Older patients were significantly less likely to frequently refresh for low-sensitivity results, suggesting that refresh behavior may be associated with access to or familiarity with technology.

Many racial categories were not significantly associated with refresh activity. There are several possible explanations for these findings. Despite our corpus of more than 1 million tests, there may not have been a large enough sample of some subpopulations in our cohort. It is possible that the tendency to refresh while awaiting results is a patient characteristic that does not differ widely at the population level.

There are multiple reasons for why individuals might refresh for results, including being worried, proactive about their care, or highly engaged in self-management. Research is necessary to validate these patterns. We also observed that patients who enabled notifications for results had higher odds of frequent refreshing. During this study, VUMC turned off default notifications for new results.^3^ We hypothesized that patients who enable notifications may be more experienced in using the portal or have greater digital literacy. Alternatively, these patients may be more vigilant, anxious, or proactive about their care.

Measuring refresh activity provides insights into patterns that may contribute to increased messaging. Refresh for low-sensitivity results, compared with high-sensitivity results, had nearly 2.5 times the change for messaging. For result refreshing in the fifth percentile, this change corresponded to a 7.2% probability of messaging associated with refresh behavior after reviewing low-sensitivity results compared with a 2.9% probability of messaging for high-sensitivity results. There are several possible explanations for this finding. First, refresh behavior about these frequently ordered tests may represent individual preference, rather than a result characteristic, that may contribute most substantially to message-related workload. Second, benign results for high-sensitivity tests may decrease motivation to send a message, and in contrast, low-sensitivity tests may be difficult to interpret and contextualize. Tests with numeric results, including a disproportionate number of low-sensitivity tests, are commonly presented with a reference range or indicator of abnormality without additional context. The design and display of quantitative results, including accompanying educational content, are important directions for future work that may decrease messaging. Third, the finding may also reflect differences in precounseling workflows that are often in place for highly sensitive results, such as those that might indicate cancer. A prior multisite study found that precounseling was associated with lower odds of worry at some sites.^7^ Research is needed to understand best practices in precounseling and the association of precounseling with patient worry and messaging.

Our findings have policy implications that should be considered when developing strategies to improve result release and review. In response to clinician concerns, several states proposed or passed legislation to block immediate availability of sensitive results, such as those related to cancer diagnosis.^26,27,28^ This legislation was intended to limit risk of patients learning of a life-altering diagnosis without professional guidance, misinterpreting laboratory results, and reducing the influx of messages. Our findings support that at a population level, sensitive results themselves may not be a primary reason for refresh activity and patient messaging, a possible surrogate measure of worry. Patient characteristics, attitude, and preferences may contribute more substantially to feelings of worry or anxiety about results.^7,29^ Current vendor electronic health record systems offer limited flexibility to incorporate patient preference into result release workflows. Policy makers, health systems, and electronic health record vendors should consider expanding opportunities that allow patients to receive and review results according to their preferences at a granular level.

### Limitations

This study has several limitations. First, we conducted our analysis using data from outpatient test results released at a single site that used a patient portal. Patients enrolled in the portal at VUMC are disproportionally female, White, non-Hispanic, and English-speaking individuals.^2^ The generalizability of our findings needs to be confirmed in other organizations, patient populations, and patient portals. Second, this cross-sectional study was retrospective, and there may have been additional confounding variables not addressed. Third, we could not pinpoint which tests specifically contributed to refresh behaviors because we collapsed results to 6-hour windows. However, by analyzing results within the same sensitivity class, we were able to minimize potential contamination. Finally, we measured patient-initiated messaging relative to the timing of result review without analysis of message content.

## Conclusions

This cross-sectional study quantified refresh behavior while awaiting test results in the patient portal. We observed that patients who were younger, non-Hispanic, and English-speaking individuals and who manually enabled notifications for new results had the greatest odds of exhibiting frequent refresh behavior. We found that patients who refreshed for low-sensitivity results had a nearly 2.5-times greater odds of sending a message after reviewing their result compared with patients who refreshed for highly sensitive results. These findings suggest that refresh behavior while awaiting new test results may be a characteristic of individual patient attitudes and preferences rather than the test itself. As health systems balance result availability while limiting undue patient worry and increased clinical workload, there remains an opportunity to tailor result release strategies to individual patient attitudes and preferences.

## Notes

Supplement 1. **eTable 1.** Example Top 20 Low-Sensitivity and High-Sensitivity Tests by Order Volume **eTable 2. **Demographic Characteristics of Patients Who Received and Reviewed Test Results Via Portal, Stratified by Result Sensitivity **eTable 3. **Unadjusted Marginal Effects of Refresh Activity on Patient-Initiated Messaging Within 24 Hours of Result Review

Supplement 2.
Data Sharing Statement
