# Supplementary material for: Repeated Access to Patient Portal While Awaiting Test Results and Patient-Initiated Messaging
Source: JAMA Netw Open. 2025 Apr 8;8(4):e254019. doi: 10.1001/jamanetworkopen.2025.4019 (PMC11979724; doi:10.1001/jamanetworkopen.2025.4019)
Supplement: Supplement 1. — eTable 1. Example Top 20 Low-Sensitivity and High-Sensitivity Tests by Order Volume eTable 2. Demographic Characteristics of Patients Who Received and Reviewed Test Results Via Portal, Stratified by Result Sensitivity eTable 3. Unadjusted Marginal Effects of Refresh Activity on Patient-Initiated Messaging Within 24 Hours of Result Review [file jamanetwopen-e254019-s001.pdf]

Supplemental Online Content

Steitz BD, Turer RW, Salmi L, et al. Repeated access to patient portal while awaiting test results and patient-initiated messaging. *JAMA Netw Open*. 2025;8(4):e254019. doi:10.1001/jamanetworkopen.2025.4019

eTable 1. Example Top 20 Low-Sensitivity and High-Sensitivity Tests by Order Volume
eTable 2. Demographic Characteristics of Patients Who Received and Reviewed Test Results Via Portal, Stratified by Result Sensitivity
eTable 3. Unadjusted Marginal Effects of Refresh Activity on Patient-Initiated Messaging Within 24 Hours of Result Review

This supplemental material has been provided by the authors to give readers additional information about their work.

© 2025 Steitz BD et al. *JAMA Network Open*.

eTable 1. Example Top 20 Low-Sensitivity and High-Sensitivity Tests by Order Volume

Test sensitivity was determined using historic release categories that guided result release timing prior to the Cures Act.

**Low Sensitivity Tests (Historic Immediate Release)**	**High Sensitivity Tests (Historic 14 day Delay)**
Automated Differential Blood Count	Breast Biopsy Report
Basic Metabolic Panel	Breast Imaging Post Biopsy Mammogram
C-Reactive Protein Test	Fine-Needle Aspiration Cytology
Complete Blood Count	Flow Cytometry Leukemia/Lymphoma
Comprehensive Metabolic Panel	Flow Cytometry Report
Estimated Glomerular Filtration Rate	Gynecological Cytopathology Report
Ferritin Test	Hematopathology Body Fluid Report
Free T4	Hematopathology Bone Marrow Report
Hemoglobin A1C	Hematopathology Report
Hepatic Functional Panel	Liver Biopsy Report
Lipid Panel	Neuropathology Report
Magnesium Level	Non-Gynecologic Cytopathology Report
Microscopic Urinalysis	Paroxysmal Nocturnal Hemoglobinuria Report
Phosphorus Level	Pathology Report
Prothrombin Time	Percutaneous Biopsy Report
SARS-COV-2 PCR	Renal Pathology Report
Thyroid-Stimulating Hormone Test	Surgical Pathology Consult Report
Troponin Test	Surgical Pathology Report
Urine Culture	Thyroid Biopsy
Vitamin D Total	Urine Cytology

© 2025 Steitz BD et al. *JAMA Network Open*.

eTable 2. Demographic Characteristics of Patients Who Received and Reviewed Test Results Via Portal, Stratified by Result Sensitivity

Summary statistics are calculated per test result. Percentages are calculated by result sensitivity across rows.

	No. of patients (%)							
	Low Sensitivity				High Sensitivity			
	Never View (N=223,244)	View Without Refresh (N=678,902)	View and Refresh (N=225,516)	All Results (N=1,127,662)	Never View (N= 16,187)	View Without Refresh (N= 39,076)	View and Refresh (N= 25,280)	All Results (N=80,543)
**Age (Years)**								
18 - 34	40,953 (18.3)	160,398 (23.6)	55,843 (24.8)	257,194 (22.8)	5,387 (33.3)	15,945 (40.8)	10,548 (41.7)	31,880 (39.6)
35 - 49	38,398 (17.2)	148,575 (21.9)	52,990 (23.5)	239,963 (21.3)	4,850 (30.0)	11,200 (28.7)	7,583 (30.0)	23,633 (29.3)
50 - 64	59,920 (26.8)	180,061 (26.5)	61,780 (27.4)	301,761 (26.8)	4,119 (25.4)	8,164 (20.9)	5,075 (20.1)	17,358 (21.6)
65 - 84	77,243 (34.6)	180,088 (26.5)	52,690 (23.4)	310,021 (27.5)	1,741 (10.8)	3,623 (9.3)	2,002 (7.9)	7,366 (9.1)
85 or older	6,730 (3.0)	9,780 (1.4)	2,213 (1.0)	18,723 (1.7)	90 (0.6)	144 (0.4)	72 (0.3)	306 (0.4)
**Sex**								
Female	128,565 (57.6)	436,529 (64.3)	150,868 (66.9)	715,962 (63.5)	14,790 (91.4)	35,355 (90.5)	23,303 (92.2)	73,448 (91.2)
Male	94,679 (42.4)	242,373 (35.7)	74,648 (33.1)	411,700 (36.5)	1,397 (8.6)	3,721 (9.5)	1,977 (7.8)	7,095 (8.8)
**Ethnicity**								
Hispanic/Latino	11,550 (5.2)	32,903 (4.8)	10,044 (4.5)	54,497 (4.8)	1,106 (6.8)	2,301 (5.9)	1,526 (6.0)	4,933 (6.1)
Not Hispanic/Latino	185,488 (83.1)	576,455 (84.9)	195,722 (86.8)	957,665 (84.9)	13,363 (82.6)	33,310 (85.2)	21,812 (86.3)	68,485 (85.0)
Other/Unknown[a]	26,206 (11.7)	69,544 (10.2)	19,750 (8.8)	115,500 (10.2)	1,718 (10.6)	3,465 (8.9)	1,942 (7.7)	7,125 (8.8)
**Race**								
American Indian or Alaska Native	774 (0.3)	2,694 (0.4)	963 (0.4)	4,431 (0.4)	67 (0.4)	144 (0.4)	88 (0.3)	299 (0.4)
Asian	3,003 (1.3)	13,517 (2.0)	4,592 (2.0)	21,112 (1.9)	332 (2.1)	894 (2.3)	702 (2.8)	1,928 (2.4)
Black or African American	39,172 (17.5)	80,885 (11.9)	21,939 (9.7)	141,996 (12.6)	1,955 (12.1)	3,851 (9.9)	3,083 (12.2)	8,889 (11.0)
Middle Eastern or North African	723 (0.3)	2,246 (0.3)	679 (0.3)	3,648 (0.3)	66 (0.4)	108 (0.3)	88 (0.3)	262 (0.3)
Native Hawaiian or Other Pacific Islander	193 (0.1)	587 (0.1)	175 (0.1)	955 (0.1)	13 (0.1)	36 (0.1)	21 (0.1)	70 (0.1)
White	162,373 (72.7)	533,001 (78.5)	184,051 (81.6)	879,425 (78.0)	11,909 (73.6)	30,742 (78.7)	19,164 (75.8)	61,815 (76.7)
Other/Unknown[b]	17,006 (7.6)	45,972 (6.8)	13,117 (5.8)	76,095 (6.7)	1,845 (11.4)	3,301 (8.4)	2,134 (8.4)	7,280 (9.0)
**Preferred Language**								
English	215,399 (96.5)	667,697 (98.3)	223,041 (98.9)	1,106,137 (98.1)	15,376 (95.0)	38,347 (98.1)	24,761 (97.9)	78,484 (97.4)
Spanish	4,515 (2.0)	5,536 (0.8)	1,190 (0.5)	11,241 (1.0)	455 (2.8)	393 (1.0)	293 (1.2)	1,141 (1.4)
Other Language	3,330 (1.5)	5,669 (0.8)	1,285 (0.6)	10,284 (0.9)	356 (2.2)	336 (0.9)	226 (0.9)	918 (1.1)
**Insurance**								
Commercial	102,607 (46.0)	407,107 (60.0)	140,828 (62.4)	650,542 (57.7)	11,435 (70.6)	30,235 (77.4)	19,228 (76.1)	60,898 (75.6)
Medicaid	12,478 (5.6)	31,562 (4.6)	9,939 (4.4)	53,979 (4.8)	1,784 (11.0)	3,096 (7.9)	2,547 (10.1)	7,427 (9.2)
Medicare	51,702 (23.2)	122,263 (18.0)	39,708 (17.6)	213,673 (18.9)	1,171 (7.2)	2,530 (6.5)	1,499 (5.9)	5,200 (6.5)
Uninsured	6,058 (2.7)	11,968 (1.8)	3,093 (1.4)	21,119 (1.9)	218 (1.3)	481 (1.2)	257 (1.0)	956 (1.2)
Other[c]	50,399 (22.6)	106,002 (15.6)	31,948 (14.2)	188,349 (16.7)	1,579 (9.8)	2,734 (7.0)	1,749 (6.9)	6,062 (7.5)
**Years Enrolled in Portal**								
Mean (SD)	2.0 (1.8)	2.3 (1.8)	2.4 (1.8)	2.3 (1.8)	2.0 (1.9)	2.1 (1.8)	2.1 (1.8)	2.1 (1.8)
Median [Q1, Q3]	1.7 [0.0, 4.2]	2.2 [0.4, 4.2]	2.5 [0.5, 4.2]	2.1 [0.4, 4.2]	1.7 [0.0, 4.0]	1.9 [0.2, 4.1]	1.8 [0.1, 4.2]	1.8 [0.1, 4.1]
**Notified of Result?**								
No	176,573 (79.1)	380,074 (56.0)	93,907 (41.6)	650,554 (57.7)	11,487 (71.0)	16,291 (41.7)	8,990 (35.6)	36,768 (45.7)
Yes	46,671 (20.9)	298,828 (44.0)	131,609 (58.4)	477,108 (42.3)	4,700 (29.0)	22,785 (58.3)	16,290 (64.4)	43,775 (54.3)
**Patient-Initiated Message within 24 hours**								
No	208,886 (93.6)	583,366 (85.9)	175,799 (78.0)	968,051 (85.8)	15,706 (97.0)	35,623 (91.2)	22,016 (87.1)	73,345 (91.1)

© 2025 Steitz BD et al. *JAMA Network Open*.

Yes	14,358 (6.4)	95,536 (14.1)	49,717 (22.0)	159,611 (14.2)	481 (3.0)	3,453 (8.8)	3,264 (12.9)	7,198 (8.9)

[a] Includes “Unknown”, “Prefer Not to Answer”, or “Unable to Provide” as indicated in the EHR.
[b] Includes “None of These”, “Other”, “Prefer Not to Answer”, and “Unable to Provide” as indicated in the EHR
[c] Includes workers’ compensation and nonclassified payers

eTable 3. Unadjusted Marginal Effects of Refresh Activity on Patient-Initiated Messaging Within 24-hours of Result Review

	AME (95 CI)	*P* value
All Categories	0.98 (0.92-1.04)	<.001
Low Sensitivity	1.14 (1.07-1.21)	<.001
High Sensitivity	0.41 (0.34-0.48)	<.001

© 2025 Steitz BD et al. *JAMA Network Open*.
