# Supplementary material for: Repeated Access to Patient Portal While Awaiting Test Results and Patient-Initiated Messaging
Source: JAMA Netw Open. 2025 Apr 8;8(4):e254019. doi: 10.1001/jamanetworkopen.2025.4019 (PMC11979724; doi:10.1001/jamanetworkopen.2025.4019)
Supplement: Supplement 2. — Data Sharing Statement [file jamanetwopen-e254019-s002.pdf]

Data Sharing Statement

Steitz. Repeated Access to Patient Portal While Awaiting Test Results and Patient-Initiated Messaging. *JAMA Netw Open*. Published April 08, 2025.
doi:10.1001/jamanetworkopen.2025.4019

Data
Data available: No

Additional Information
Explanation for why data not available: The data collected for this study contain numerous patient and clinician identifiers, which we are prohibited from sharing.
